# Viral Parkinsonism: An underdiagnosed neurological complication of Dengue virus infection

**DOI:** 10.1371/journal.pntd.0010118

**Published:** 2022-02-09

**Authors:** Hannah K. Hopkins, Elizabeth M. Traverse, Kelli L. Barr

**Affiliations:** Center for Global Health and Infectious Disease Research, University of South Florida, Tampa, Florida, United States of America; KU Leuven, BELGIUM

## Abstract

Dengue virus (DENV) is a flavivirus that is a significant cause of human disease costing billions of dollars per year in medical and mosquito control costs. It is estimated that up to 20% of DENV infections affect the brain. Incidence of DENV infections is increasing, which suggests more people are at risk of developing neurological complications. The most common neurological manifestations of DENV are encephalitis and encephalopathy, and movement disorders such as parkinsonism have been observed. Parkinsonism describes syndromes similar to Parkinson’s Disease where tremors, stiffness, and slow movements are observed. Parkinsonism caused by viral infection is characterized by patients exhibiting at least two of the following symptoms: tremor, bradykinesia, rigidity, and postural instability. To investigate DENV-associated parkinsonism, case studies and reports of DENV-associated parkinsonism were obtained from peer-reviewed manuscripts and gray literature. Seven reports of clinically diagnosed DENV-associated parkinsonism and 15 cases of DENV encephalitis, where the patient met the case criteria for a diagnosis of viral parkinsonism were found. Clinically diagnosed DENV-associated parkinsonism patients were more likely to be male and exhibit expressionless face, speech problems, and lymphocytosis. Suspected patients were more likely to exhibit tremor, have thrombocytopenia and low hemoglobin. Viral parkinsonism can cause a permanent reduction in neurons with consequential cognitive and behavior changes, or it can leave a latent imprint in the brain that can cause neurological dysfunction decades after recovery. DENV-associated parkinsonism is underdiagnosed and better adherence to the case definition of viral parkinsonism is needed for proper management of potential sequalae especially if the patient has an ongoing or potential to develop a neurodegenerative disease.

## Author summary

Dengue Virus (DENV) causes generalized fever in most patients and is transmitted via *Aedes aegypti* mosquitos. A small proportion of DENV infected patients have neurological complications associated with the critical phase of the illness. The usual neurological manifestations are encephalitis and encephalopathy, but there can also be movement disorders such as parkinsonism. DENV patients with parkinsonism present with tremor, bradykinesia, instability, and rigidity on top of the typical febrile manifestations of the disease. We searched the literature and uncovered 7 cases of clinically diagnosed DENV parkinsonism patients and 15 cases of suspected DENV parkinsonism. We found that the clinically diagnosed patients were more likely to be male, have expressionless face, speech issues and lymphocytosis. The suspected cases often had a diagnosis of encephalitis and were more likely to have tremors, thrombocytopenia, and low hemoglobin.

## Introduction

Dengue virus (DENV) is a flavivirus that is a significant cause of human disease [1–3]. It is spread by the mosquitos *Aedes aegypti* and *Aedes albopictus* and humans are a crucial part of the viral life cycle [1,2,4,5]. The virus is endemic in over 100 nations and is a persistent cause of human infection with about 100 million people developing a clinically apparent DENV infection and about 22,000 people dying every year [5–7]. Additionally, the financial burden of the disease is significant, costing billions of dollars per year in medical and mosquito control costs [2,3,5,6].

Disease caused by a DENV infection is of concern because of the lasting effects it can have on those infected. DENV infection can be broken into two categories: dengue and severe dengue [5–7]. Dengue typically presents as a self-limited febrile illness, while severe dengue can result in plasma leakage, organ impairment and hemorrhagic fever [7]. Severe dengue also includes any DENV infection resulting in neurological manifestations [7]. Once thought to be non-neurotropic, it is now estimated that anywhere from 0.5% to 20% of DENV infections affect the brain [8–13]. Neurologic complications of DENV were first described in 1945 by a doctor in the Central Pacific who described 13 of his patients as having “unilateral attacks of neuritis” including the “facial, palatine, long thoracic, ulnar, peroneal and sciatic nerves” [14]. These observations were not reported again until much later in 1976 [8–15]. The most common neurological manifestations of severe dengue are encephalitis and encephalopathy, while movement disorders such as parkinsonism have been observed [8,16–32].

Parkinsonism is a term used to describe syndromes similar to Parkinson’s Disease where tremors, stiffness, and slow movements are observed [33]. Parkinsonism caused by viral infection is characterized by patients exhibiting at least two of the following symptoms: tremor, bradykinesia, rigidity, and postural instability [34]. Several viruses cause parkinsonism and regardless of the virus, there are some common symptoms that may appear including bradykinesia, tremor, hypokinesia, mask-like facies, tremor, postural instability, rigidity, and abnormal gait [35]. A study of two flaviviruses, Japanese Encephalitis Virus (JEV) and West Nile Virus (WNV), found that parkinsonism improved over time, though patients with reoccurring JEV infection had a higher rate of developing parkinsonism [35]. Many viruses including: influenza A, coxsackie virus, HIV, and many flaviviruses including JEV, St. Louis Encephalitis Virus, and WNV can cause primary or secondary parkinsonism, including encephalitis lethargica[33,36,37]

A growing body of evidence is indicating that viruses that cause parkinsonism are also associated with idiopathic Parkinson’s Disease (PD) [35,38]. PD is lifelong, characterized by neuronal death, and has about a 15-year life expectancy after the onset of symptoms [33]. Around 10% of PD cases can be attributed to a genetic etiology, while the other 90% are categorized as sporadic or idiopathic PD [33,39]. Like PD, parkinsonism is associated with reduced dopamine production resultant from neuron damage [40,41]. In addition, patients with viral parkinsonism often have cell damage that inhibits the production and uptake of dopamine[42]. Thus, treatment with levodopa may not be helpful in ameliorating symptoms [33]. Viral parkinsonism is theorized to be a result of immune mediated complications resulting in neuroinflammation even though in many cases, CT and MRI are normal [24,43]. Recent work has shown that blockage of the IFN1 pathway causes mitochondrial dysfunction in the brain which causes neuronal death [38]. Many viruses, including DENV, block IFN1 in order to evade the immune system and establish infection [44,45]. It is theorized that viral activation of microglia causes hypercytokinemia and aggregation of microglia around dopa neurons which then leads to a state of chronic inflammation and neuronal damage [46]. Viruses can also induce more neuronal damage via replication and cell lysis [47,48].

While viruses can cause acute neuroinflammation and parkinsonism, little attention has been given to long-term sequalae following recovery from the acute phase of infection. Upon immune clearance of virus from the brain, there can be a permanent reduction in neurons with consequential cognitive and behavior changes in the patient [49,50]. Many viruses can be found residing in the brain for months following recovery from the acute phase of disease in the absence of neurological symptoms [51–53]. And some viruses, like polio and WNV, have been shown to leave a “latent imprint” in the brain that causes neurological dysfunction decades after recovery [49,54,55].

Incidence of DENV infections is increasing, which suggests more people are at risk of developing neurological complications [5,56]. Viral parkinsonism caused by DENV infection may be more common than previously thought. The mechanism of DENV infection in the brain is still largely unknown, but it is theorized that damage can occur either by direct viral invasion, through an autoimmune response, or through metabolic disturbances [8,57].The likeliest route of entry is the blood brain barrier (BBB), which is a series of brain capillaries essential for the transport of oxygen and nutrients to neurons [58]. These capillaries are lined by endothelial cells that control permeability between the vascular space and parenchyma [58]. It is proposed the DENV most likely invades the brain though inter-endothelial tight junction disruption, endothelial cell infection and passage through infected monocytes [58].

In order to investigate DENV-associated parkinsonism, case studies and reports of DENV-associated parkinsonism were obtained from peer-reviewed manuscripts and gray literature. Clinical features and demographics were analyzed to determine if any hallmark features were associated with the diagnosis of parkinsonism.

## Methods

### Case definition

Inclusion criteria for DENV-associated parkinsonism were as follows: a clinical diagnosis of a DENV infection with laboratory confirmation and a diagnosis of parkinsonism either during or immediately following the acute phase of infection. Patients with a clinical diagnosis of a DENV infection and at least two of the diagnostic symptoms of parkinsonism (bradykinesia, tremor, hypokinesia, mask-like facies, tremor, postural instability, rigidity, and abnormal gait) either during or immediately following the acute phase of infection but no officially diagnosed parkinsonism, were included in a separate group since they met criteria for a clinical diagnosis [33,34].

#### Search Strategy

Manuscripts and case reports were obtained from Pubmed, Scopus, CINAHL, Embase, LILACs, Google Scholar, and gray literature from medical and government agencies. The search terms used to find cases were “dengue + Parkinson” and “dengue + parkinsonism.” In order to find suspected case reports of dengue parkinsonism, infections with overlapping symptoms, the search terms used were: “dengue + tremor,” “dengue + bradykinesia,” “dengue + rigidity,” “dengue + instability,” and “dengue + encephalitis + case.”

### Statistical analysis

Patients were divided into two groups which included: patients with viral parkinsonism as a primary diagnosis (Clinical Cases) and patients with symptoms of viral parkinsonism but lacking a clinical diagnosis of parkinsonism (Suspect Cases). Clinical laboratory values were compared against the normal reference range for the appropriate sex as needed (i.e. hematocrit). Statistical analyses were performed using MedCalc version 17.9.7–64-bit. Logistic regression for dichotomous independent variables was performed. Odds ratios were calculated with 95% confidence intervals. Ratios with a p<0.05 were considered significant. When the odds ratios were less than 1, the binary values in the analysis were reversed to obtain the inverse odds ratio. When the 95% CI was not calculated by the software due to small sample size, p-values were reported. When appropriate, ANOVA with Tukey-Kramer post-hoc test was performed.

## Results

### Dengue parkinsonism

**Fig 1:**
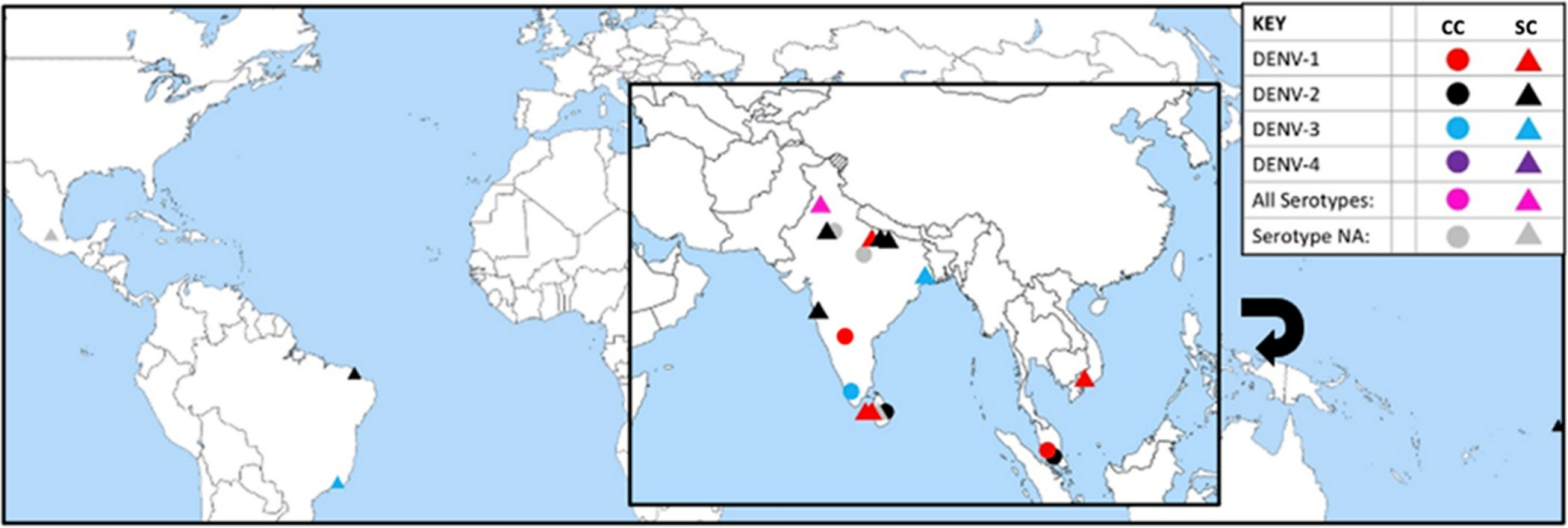
Locations of clinical cases of DENV parkinsonism (CC) and suspect cases (SC). Only cases with location available were included. Dot location is approximate and there is location overlap for some cases. Serotype was inferred through sequence submissions to NCBI and peer-reviewed outbreak reports from locations where and when these cases occurred. The base layer of this map is from (https://commons.wikimedia.org/wiki/File:A_large_blank_world_map_with_oceans_marked_in_blue.svg) by Petr Dlouhý, July 25, 2006. Wikimedia Commons.

The literature search produced 1,990 sources though when examined, most reports did not include sufficient data to determine if the patient had parkinsonism. Thus, only seven case reports of clinically diagnosed DENV-associated parkinsonism were found and included for analysis (Table 1). The first reported case occurred in 2013 in Kuala Lumpur, Malaysia and after reports originated from Kuala Lumpur, Malaysia; Kandy, Sri Lanka; Calicut, India; Northern India; New Delhi India; and the Karnataka State in India (Fig 1), [24]. Many reports did not provide a serotype as patients were diagnosed via ELISA for either NS1, IgG or IgM. Thus, serotype was inferred through sequence submissions to NCBI and peer-reviewed outbreak reports from locations where and when these cases occurred. In 2013, Kuala Lumpur, Malaysia had an outbreak of DENV-1 and DENV-2 [59], while only having DENV-1 in 2014 [60]. DENV-2 was present in Kandy, Sri Lanka in 2017 [61]. Calicut, India had DENV-3 in circulation in 2019 [62]. The serotype of DENV in Northern India and New Delhi in 2020 was not able to be determined. The serotype in India in 2021 was DENV-1 based on reference sequences in the NCBI virus database [63].

**Table 1:** References for Clinically Diagnosed Cases of DENV Parkinsonism.

Clinically Diagnosed Cases
Azmin et al. 2013 [24]
Fong et al. 2014 [89]
Bopeththa et al. 2017 [88]
Manappallil et al. 2019 [90]
Panda et al. 2020 [93]
Mishra et al. 2020 [92]
Ganraja et al. 2021 [91]

Searches for DENV with suspected parkinsonism produced 544 papers. Here too, most authors failed to include sufficient information to determine if the patient had parkinsonism. Thus, only 15 cases met the inclusion criteria for DENV with viral parkinsonism (Table 2). Cases were identified from India, Brazil, Mexico, Sri Lanka, Vietnam, and Tahiti (Fig 1). Most cases did not report serotype, however the serotypes of many of the other case studies were inferred as described above. Five reports were associated with outbreaks of DENV-2 [64–73]. Three of the case studies were associated with outbreaks of DENV-1 [74–77]. Two were associated with DENV-3 and none with DENV-4 [78–81]. One case was in a region with all four serotypes present, and two in a region where DENV-1 and DENV-2 were co-circulating [82–87].

**Table 2:** References for the Suspected Cases of DENV Parkinsonism.

Suspected Cases of Parkinsonism
Vasconcelos et al. 1998 [65]
Palma da Cunha Matta et al. 2004 [80]
Shah 2007 [64]
Khanna et al. 2011 [67]
Verma et al. 2011 [86]
Karunarathne et al. 2012 [74]
Samanta et al. 2012 [78]
Withana et al. 2014 [76]
Verma et al. 2016 [68]
Doi et al. 2017 [69]
Pal et al. 2017 [82]
Osnaya-Romero et al. 2017 [95]
Nguyen et al. 2018 [84]
Weerasinghe and Medagama 2019 [94]
Johnson et al. 2019 [75]

### Patient demographics

**Fig 2:**
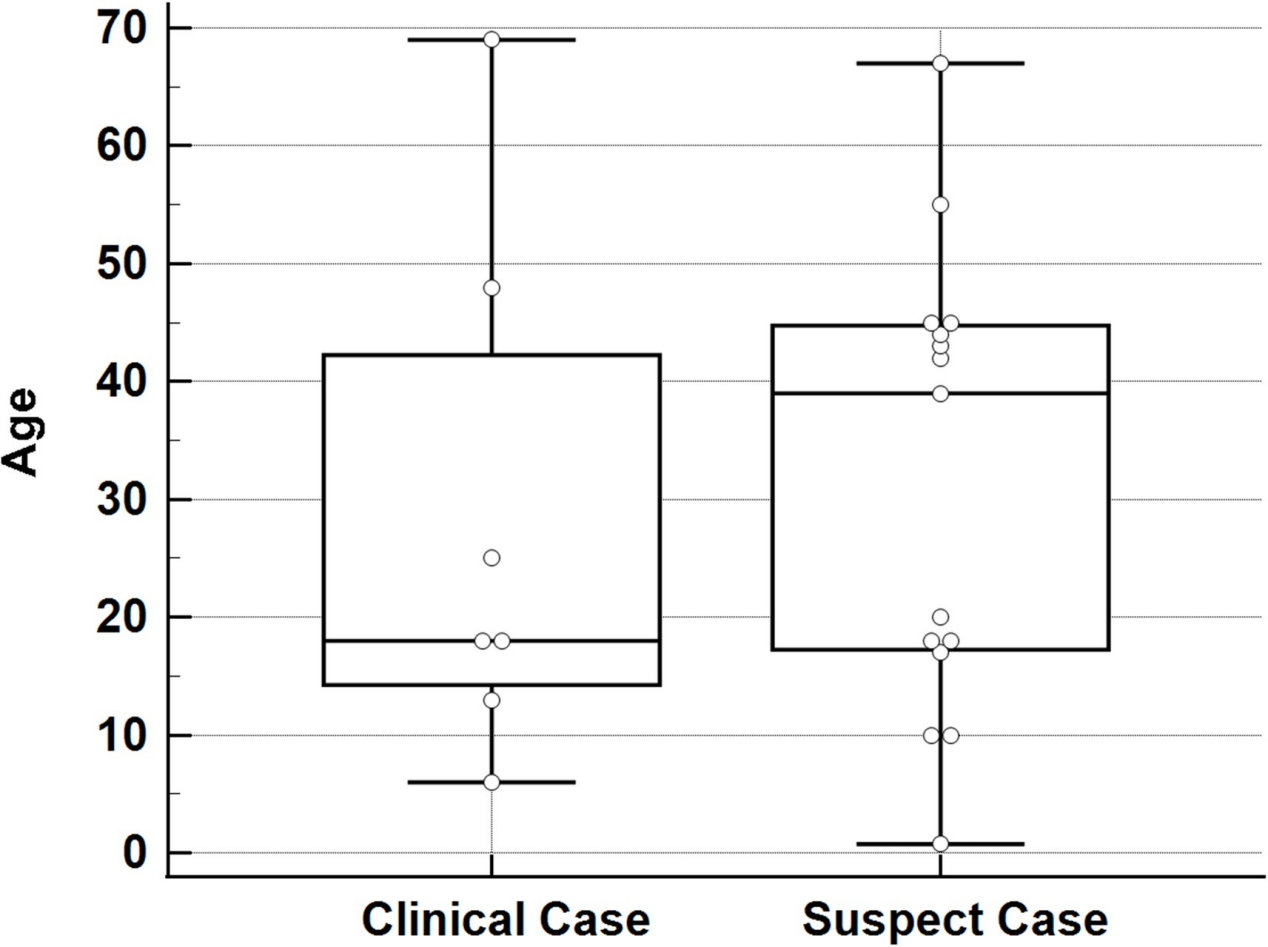
Age distribution of patients with clinical and suspect dengue-associated parkinsonism. The center line indicates the median value (50^th^ percentile), while the box contains the 25^th^ to the 75^th^ percentiles. Error bars represent 95% confidence interval.

The average age of patients with a clinical diagnosis of parkinsonism was 28.14 years (SD +/- 22.36) with a range of 63 years (Table 3, Fig 2). Five of the 7 cases were aged 25 or younger while one was aged 48 and the oldest was 69 (Fig 2). 85.71% were male and 14.29% female (Table 3). Two of the six cases had a significant medical history: one had chicken pox two months prior to DENV infection [24] and another had a history of non-Hodgkin’s lymphoma [88]. For suspect cases, 46% were male and 54% female (Table 3). The average patient age was 31.58 (SD +/- 19.30) with a range of 66.25 years (Table 3, Fig 2). Here, 7 of the patients were aged 25 or younger while 6 were between the ages of 39 to 45 and the 2 remaining patients were aged 55 and 67 (Fig 2). Of note, there were no patients between 26 and 42 years of age. Patients with a clinical diagnosis of parkinsonism were 6.85 times more likely to be male than patients without a clinical diagnosis of parkinsonism (Table 3).

**Table 3:** Patient demographics. OR = odds ratio, CI = confidence interval, NS = not significant.

Variable	Clinical Case	Suspect Case	OR (95% CI)
Males/Females	6/1 (86%)	7/8 (46%)	6.85 (0.65 to 71.72)
Age	28.14 (6–69)	31.58 (0.75–67)	NS

### Vital statistics

Heart rate and blood pressure were within normal range for both groups (Table 4). Both groups had an average temperature over 38°C (Table 4). For both groups of patients, vital statistics were not a significantly different from each other (p >0.1) (Table 4).

**Table 4:** Vital statistics for patients with clinical and suspect dengue-associated parkinsonism. Bpm = beats per minute, mmHg = millimeters of mercury.

	Clinical Case	Suspect Case	P-value
Heart rate (bpm)	96.67 (80–110)	92 (72–104)	>0.1
Systolic (mmHg)	120 (110–130)	121.44 (107–150)	>0.1
Diastolic (mmHg)	73.33 (70–80)	75.11 (60–100)	>0.1
Temperature (°C)	38.95 (38.3–40)	38.48 (37.5–40)	>0.1

### Clinical manifestations

For patients with a clinical diagnosis of parkinsonism, symptom onset occurred immediately after or concurrently with the critical phase of a severe dengue fever [88–91]. Additionally, three of the seven clinically diagnosed patients in the studies tested positive for IgG which indicates a likely secondary infection [24,89,92]. No patient deaths were reported [24,88–91,93], but one outcome was not described [92]. All patients with a diagnosis of parkinsonism also had severe DENV as defined by the World Health Organization [7]. Symptoms for the clinical case group were expressionless face in 5 of 7 cases (Table 5), [88,89,91–93]. Two of seven experienced imbalance [24,93] and four of seven had confusion (Table 5), [89–92]. Of the seven case studies, six had slurring of speech (Table 5), [24,88–91,93]. Six had slow movements [24,88–91,93], four had tremors/dystonia [24,90–92] and five had gait issues (Table 5), [24,88,89,91,93]. Four case studies reported stiffness/rigidity (Table 5), [88,90,91,93]. The clinical case group was 16.25 times more likely to exhibit expressionless faces than the suspect group (Table 5). In the clinical case group, speech problems were 6.86 times more likely to be reported, slow movements were 4 times likely to be reported, and encephalitis was equally likely to be diagnosed than the suspect group (Table 5). Levodopa was prescribed in 6 of the 7 clinical cases and 2 out of 4 suspect cases where prescribing information was included. While levodopa was 6 times more likely to be used following clinical diagnosis of parkinsonism, it was only 60% vs 50% effective in the symptomatic group (Table 5).

**Table 5:** Clinical features of patients with clinical parkinsonism and suspect parkinsonism.

	Clinical Case	Suspect Case	OR (95% CI)
	*N* (%)	*N* (%)	
Rash	2 (29%)	3 (20%)	1.6 (0.2 to 12.69)
Hemorrhagic fever	2 (29%)	3 (20%)	1.2 (0.15 to 9.76)
Fever ≥38°C	7 (100%)	12 (80%)	P = 0.1778
Expressionless face	5 (71%)	2 (13%)	16.25 (1.77 to 148.84)
Imbalance	2 (29%)	7 (47%)	2.19 (0.31 to 15.04)*
Confusion	4 (57%)	7 (46%)	1.67 (0.19 to 7.11)
Speech problems	6 (86%)	7 (47%)	6.86 (0.65 to 71.72)
Weakness	1 (17%)	3 (20%)	0.63 (0.08 to 4.96)*
Slow movements	6 (86%)	9 (75%)	4.0 (0.37 to 42.17)
Tremor/dystonia	4 (57%)	12 (80%)	3.0 (0.42 to 21.29)*
Problems with gait	5 (71%)	7 (46%)	2.86 (0.41 to 19.64)
Rigidity	4 (57%)	7 (46%)	1.52 (0.25 to 9.29)
Encephalitis	4 (57%)	9 (75%)	1.0 (0.13 to 7.45)*
Abnormal CSF	3 (50%)	7 (58%)	2.33 (0.33 to 16.18)*
Abnormal MRI	3 (50%)	9 (81%)	3.36 (0.39 to 28.75)*
Levadopa Used	6 (86%)	2 (13%)	6.00 (0.33 to 107.42)
Levadopa Effective	3 (43%)	1 (50%)	1.0 (0.04 to 24.54)
Death	0	5 (33%)	P = 0.0016
Serotype	1,2,3	1,2,3,4	P = 0.855
Hyponatremia	0	2 (15%)	P = 0.189
Secondary Infection	3 (50%)	7 (58%)	P = 0.1377

OR = odds ratio, CI = confidence interval, NS = not significant

* = Odds Ratio in favor of group with suspect parkinsonism.

Patients in the suspect case group, were 2.19 times more likely to experience imbalance and 3.0 times more likely to have tremor than the clinical group (Table 5). Additionally, they were 2.33 times more likely to report abnormal CSF and 3.36 times more likely to have an abnormal MRI (Table 5). Infecting serotype was not a significant factor for either group (Table 5). However, death was significantly more likely to occur in the suspect group (p = 0.0016) (Table 5).

The major neurological symptoms were varied for suspect cases. Slow movements were reported in 9 out of the 15 cases (Table 5), [67,68,74–76,78,82,86,94]. These patients exhibited slow movements and bradykinesia, an “akinetic state” and reduced spontaneity of movement, and dysdiadochokinesia (Table 5), [67,68,74–76,78,82,86,94]. Slurring or disruption of speech was also common (Table 5), [67,68,74,76,80,82,84]. Seven of the case reports describe gait instability, ataxia, motor incoordination or postural instability (Table 5), [64,67,68,74,76,80,82].

Tremors, myoclonic movements, tonic-clonic movements, generalized convulsion, clonic flexion jerks, involuntary movements, involuntary twitching, or spasticity were reported in 12 of the cases (Table 5), [64,65,67,69,75,78,80,82,84,86,94,95]. Additionally, patients exhibited cogwheel rigidity, mask-like facies, increased muscle tone, rigidity, quadriplegia, and involuntary muscle tightening (Table 5), [67–69,82,84,86,95]. Of these cases, 33.33% perished (Table 5), [65,75,78,86,95].

### MRI manifestations

MRI findings were normal for all but three patients with a clinical diagnosis of parkinsonism. One of which had microinfarcts of the basal ganglia [90] and the other had a double-doughnut sign that is typical of dengue encephalitis [92]. Another patient had brain atrophy [91]. CSF was also normal for all but three patients in this group, with one having reported lymphocytopenia, protein (58 mg/dL) [88] and another with lymphocytic pleocytosis with elevated protein (296 mg/dL) [92]. The third had abnormally high CSF total protein levels (557 mg/L) [24].

For the suspect cases, nine reported abnormal MRI’s. One reported bilaterally symmetrical T2/FLAIR hyperintensities in the caudate and lentiform nuclei [82]. Another described the young girl having “inflammatory changes” in her right maxillary, ethmoid, and sphenoid sinuses [64]. Another described their patient as having bilateral intensities in the lentiform nuclei, external capsule, and pons [67]. An additional case had hyperintense areas in the cerebellar hemisphere [74] and another reported high intensity in the subcortical white matter and cortical gray matter with some meningeal enhancement in the front temporal area [94]. A different patient had a bilateral symmetrical high signal with periventricular and deep cerebral white matter [84]. Another patient had white matter paucity in the left high parietal area [78]. Another case study reported their patient as having diffuse hyperintensities in the brainstem and cerebellum [68]. While another case report depicted the patient as having hypersignal in the T2/FLAIR sequence of the basal nuclei, cerebral penduncle and internal capsule [80].

### Hematology

Aspartate aminotransferase was not a significant feature between the 2 groups, but the clinical cases were 2.5 times more likely to have lower alanine aminotransferase (Table 6). Clinical cases were also 8.0 times more likely to have lower lymphocyte count (Table 6). Conversely, suspect cases were 1.33 times more likely to exhibit thrombocytopenia and 2.5 times more likely to have decreased hemoglobin (Table 6).

**Table 6:** Hematological profiles of patients with clinical parkinsonism and suspect parkinsonism.

	Clinical Case		Suspect Case		
	Mean (range)	*N* (%)	Mean (range)	*N* (%)	*OR (95% CI)*
AST (IU/L)	NR	NR	202 (23–820)	6 (67%)	NS
ALT (IU/L)	90 (26–170)	3 (75%)	182 (31–830)	4 (44%)	2.5 (0.16 to 38.6)
Lymphocytes (10^9^/L)	42 (64–200)	4 (80%)	72 (25–120)	1 (11%)	8.0 (0.59 to 106.94)
Platelets (10^9^/L)	123 (64–200)	3 (42%)	127 (48–324)	9 (60%)	1.33 (0.15 to 11.50)*
Hemoglobin (gm/dL)	13.7 (10.2–15.8)	1 (33%)	13 (9–16.6)	5 (50%)	2.5 (0.16 to 38.60)*

OR = odds ratio, CI = confidence interval, NR = Not Reported, NS = not significant

* = Odds Ratio in favor of the suspect case group.

## Discussion

DENV was not recognized to cause neurotropic disease until recently [8]. Thus, there is an acknowledged gap of DENV associated neuropathologies [96]. The current theorized mechanism for DENV-associated parkinsonism is infiltration of the BBB via damage from cytokines [96]. This leads to vascularization of the barrier and permeability is increased as has been shown in mice studies with cytotoxic factor (CF) and macrophage cytotoxic factor (CF_2_) [97]. These two T-lymphocyte factors are involved in macrophage and helper T cell destruction and facilitate the release of histamine which increases vascular permeability [97]. In one review, it was reported DENV can cause damage to endothelial cells, which comprise the BBB, and other neuronal cells [58]. DENV can also induce responses of microglial cells that are susceptible to DENV regardless of serotype [4,58].

In this study, the most common neurological symptoms of the clinically diagnosed DENV parkinsonism cases were mask-like facies, imbalance, confusion, slurring of speech, slow movements, tremors/dystonia, gait issues, and stiffness/rigidity. For the suspected cases, the most common neurological symptoms were slow movements, slurring/disruption of speech, instability of gait and movement, tremors/clonic movements/involuntary movements and rigidity. The officially diagnosed cases had no fatalities, while the suspected cases had a death rate of 33.33%. Additionally, the clinical cases were mostly male, while the suspect cases were only 46% male. Parkinsonism is traditionally more prevalent in men, but this also could highlight a disparity in diagnosis in women of this condition [98]. A recent review only includes parkinsonism from DENV as a childhood complication of infection, but the data gathered here shows that it can be present in all ages (Table 3), [8].

For the clinical cases, many had odds ratios greater than one and included expressionless face, confusion, speech problems, slow movements, problems with gait, rigidity, and encephalitis. These symptoms adhere to the literature regarding other flaviviruses [35,43,99]. In other studies, both WNV and JEV patients present with rigidity, hypokinesia, facial masking, bradykinesia, and postural instability, which are consistent with the criteria for a parkinsonism diagnosis [37,44].

The suspect cases had higher odds ratios for imbalance, weakness, tremor, and abnormal CSF and MRI. The most interesting of these is that suspect patients were 3 times more likely to have a tremor reported by their doctor. This raises 2 questions, the first being, could this tremor have been the typical 4–6 Hz resting tremor seen in Parkinson’s patients? Secondly, is there a reason the doctors ruled out parkinsonism as a diagnostic possibility [98]. Another factor could be the lack of information on DENV neurotropism, viral parkinsonism, or DENV parkinsonism in adults.

Two patients had hyponatremia and their doctors attributed their parkinsonism to it rather than DENV [67,82]. Hyponatremia is common in patients with both severe dengue fever and dengue shock syndrome. This is thought to be a result of either sodium depletion or excess water production due to metabolic increase as a result of disease, renal dysfunction or inhibition of the sodium potassium pump [100,101]. Parkinsonism is sometimes reported after hyponatremia of origins other than DENV [102,103]. The role hyponatremia plays in dengue-associated parkinsonism is unknown [67]. The broad range of clinical manifestations could be leading to misdiagnosis or incorrect interpretations of symptoms. Of note, roughly half of the cases in this review had normal CSF and normal MRI’s which agrees with CSF and MRI findings for other viruses that cause parkinsonism [24,88–91,93]. The absence of DENV viral particles in the CSF of the majority of patients is not surprising given that at the time of collection, viremia would have passed.

Viral parkinsonism is difficult to diagnose. In addition to physical signs and symptoms, imaging via MRI and tests of dopamine levels can help paint a picture but still may not provide evidence for a diagnosis unless significant damage to dopaminergic neurons and reduced dopaminergic levels are found. Unfortunately, vague descriptions of neurological syndromes with the generic diagnosis of encephalitis hinders the ability to collect more data on DENV-associated viral parkinsonism. The broad range of neurological symptoms exhibited by patients may be a reason (Table 5). DENV can cause Guillan-Barré syndrome, myositis, cerebellar syndrome, neuritis, hypokalemic paralysis, and many other syndromes [8,11,20,97,104–106].

Encephalitis can often show signs of “cerebral involvement,” which includes cognitive impairment and convulsions [8,107]. Myositis is characterized by “asymmetrical limb weakness” and cerebellar syndrome by nystagmus, dysarthria, and ataxia [8,20,97,104]. These overlapping symptoms to those of parkinsonism can make diagnosis difficult.

DENV-associated viral parkinsonism is likely being underdiagnosed and thus, not being described fully in the literature. The 15 suspect cases had vague diagnoses but met the case criteria for parkinsonism outlined in this paper. Some of the cases have a questionable origin for the cause of their neurological symptoms [82], others were diagnosed into the broad category of an “encephalitis” diagnosis [64], or “cerebellitis” [74], some were grouped in with seizure disorders [95]. With this variety of symptoms reported in the case studies, a more uniform reporting of symptoms could provide a clearer picture and help with differential diagnoses. Clinicians should also consider using imaging and dopamine levels of neurological cases where at least 2 characteristics of parkinsonism are present. Though this may not be possible in resource limited areas where DENV is most prevalent.

A major limitation for this study was the incomplete reporting and description of DENV encephalitis. Many reports did not include basic clinical parameters like blood counts or metabolic tests. Further, symptom descriptions were vague such as stating “patient had a fever” without providing the temperature. Hundreds of sources were excluded as they discussed patients with encephalitis but provided no clinical information or symptom description beyond headache and fever. Further, most reports did not list any information on patient outcome and patient survival could not be quantified. The widespread lack of clinical data in clinical reports hinder population-based investigations or meta-analyses that could identify patterns for risk or potential treatments. Thus, we suggest that authors making clinical reports include, at the bare minimum: age, sex, temperature, complete blood count, complete metabolic panel, complete list of symptoms, any treatments given, imaging reports (if performed), and patient outcome.

The long-term sequelae and impacts of viral encephalopathies are understudied with most reports following patients for a year or less. In pediatric reports, children with encephalitis have higher prevalence for learning disabilities and lower IQ scores [107–109]. While sequelae have been reported in over 60% of pediatric arboviral encephalitis cases, no studies performed neurocognitive testing or evaluated the efficacy of rehabilitation therapies [110,111]. Long-term cohort studies of WNV encephalitis report unresolving cognitive and mood disorders [99,112,113]. Emerging data from COVID-19 studies show that neurological infections can induce parkinsonism and worsen Parkinson’s disease [114–118]. It is clear neurological infections can have severe and permanent impacts even if patients “recover” from the acute infection.

## Conclusions

This study showed that viral parkinsonism from DENV infection may not be an isolated occurrence. Neurological manifestations of DENV are increasing in incidence and better adherence to the case definition of parkinsonism is needed for proper management of potential sequalae especially if the patient has an ongoing or potential to develop a neurodegenerative disease [8]. The inclusion of the suspected cases in this review highlights that underdiagnosis is occurring via non-adherence to the case definition for parkinsonism. This is likely due to the lack of education on DENV parkinsonism, and the symptom overlap with other neurological complications of DENV.
